# Supplementary material for: Small Peptide-Doxorubicin Co-Assembly for Synergistic Cancer Therapy
Source: Molecules. 2020 Jan 23;25(3):484. doi: 10.3390/molecules25030484 (PMC7036863; doi:10.3390/molecules25030484)
Supplement: Supplementary file 1 [file molecules-25-00484-s001.pdf]

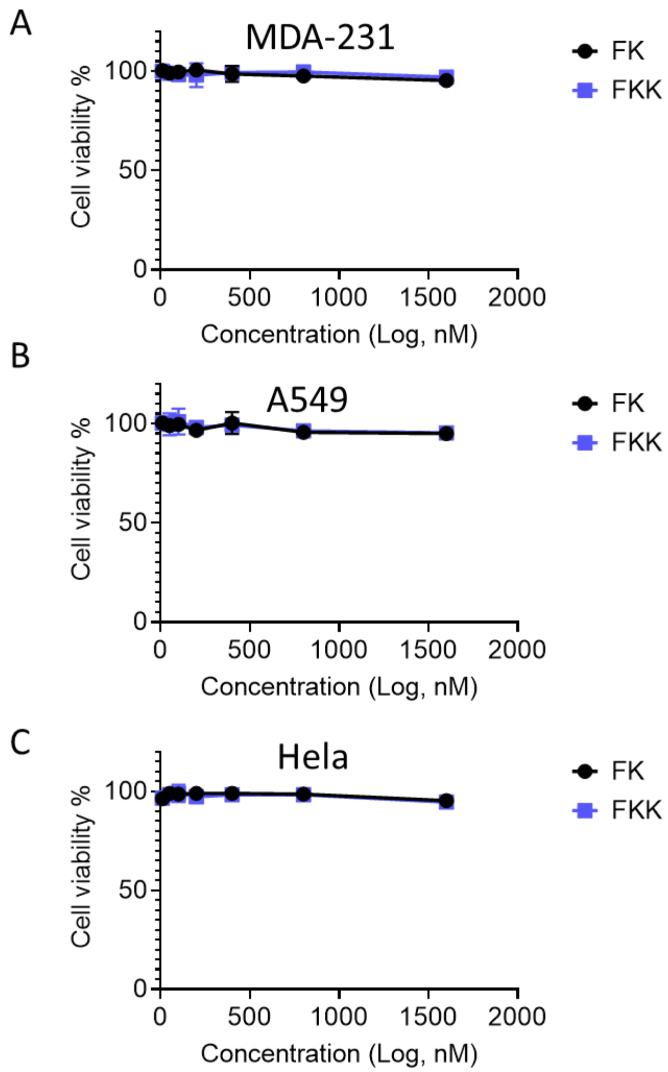

Figure S1. Cell viability measurements for MDA-231, A549, and Hela cell treated with different concentrations of FK and FKK peptides. The values were measured at 2 days post-treatment of the Dox species. Three independent replications were performed.

Measurement of ROS

The level of oxidative stress was monitored by the measurement of ROS. Hela cells from different treatment groups in the culture dishes were washed with phosphate-buffered saline (PBS) and incubated with 10 μM solution of fluorescent probe, 5-(6) chloromethyldihydroflourescein diacetate (DCFDA) (Molecular Probes, Eugene, Oregon, USA) at 37˚C for designated time in a humidified chamber. After treatment, the cells were digested and harvested from the cell culture dish and analyzed by flow cytometry. The fluorescent intensity of the cells was calculated by Flowjo software.

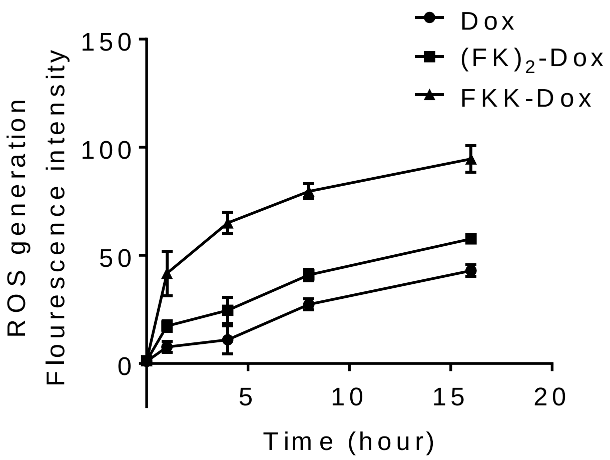

Figure S2. Levels of ROS generation in Hela cells monitored by DCFDA kit at different treating time points.
